# A study of alternative splicing in the pig

**DOI:** 10.1186/1756-0500-3-123

**Published:** 2010-05-05

**Authors:** Ann-Britt Nygard, Susanna Cirera, Michael J Gilchrist, Jan Gorodkin, Claus B Jørgensen, Merete Fredholm

**Affiliations:** 1 University of Copenhagen, Faculty of Life Sciences, Department of Basic Animal and Veterinary Sciences, Division of Genetics and Bioinformatics, Groennegaardsvej 3, 1870 Frederiksberg C, Denmark; 2 NIMR, London, NW7 1AA, UK

## Abstract

**Background:**

Since at least half of the genes in mammalian genomes are subjected to alternative splicing, alternative pre-mRNA splicing plays an important contribution to the complexity of the mammalian proteome. Expressed sequence tags (ESTs) provide evidence of a great number of possible alternative isoforms. With the EST resource for the domestic pig now containing more than one million porcine ESTs, it is possible to identify alternative splice forms of the individual transcripts in this species from the EST data with some confidence.

**Results:**

The pig EST data generated by the Sino-Danish Pig Genome project has been assembled with publicly available ESTs and made available in the PigEST database. Using the Distiller package 2,515 EST clusters with candidate alternative isoforms were identified in the EST data with high confidence. In agreement with general observations in human and mouse, we find putative splice variants in about 30% of the contigs with more than 50 ESTs. Based on the criteria that a minimum of two EST sequences confirmed each splice event, a list of 100 genes with the most distinct tissue-specific alternative splice events was generated from the list of candidates. To confirm the tissue specificity of the splice events, 10 genes with functional annotation were randomly selected from which 16 individual splice events were chosen for experimental verification by quantitative PCR (qPCR). Six genes were shown to have tissue specific alternatively spliced transcripts with expression patterns matching those of the EST data. The remaining four genes had tissue-restricted expression of alternative spliced transcripts. Five out of the 16 splice events that were experimentally verified were found to be putative pig specific.

**Conclusions:**

In accordance with human and rodent studies we estimate that approximately 30% of the porcine genes undergo alternative splicing. We found a good correlation between EST predicted tissue-specificity and experimentally validated splice events in different porcine tissue. This study indicates that a cluster size of around 50 ESTs is optimal for *in silico *detection of alternative splicing. Although based on a limited number of splice events, the study supports the notion that alternative splicing could have an important impact on species differentiation since 31% of the splice events studied appears to be species specific.

## Background

EST assembly and mapping of expressed sequences onto draft genomic sequences has resulted in large-scale detection of alternative splicing. These studies indicate that alternative splice events seem to be ubiquitous in mammalian genomes. Between one-third and two-thirds of human genes are estimated to produce at least two alternatively spliced isoforms [1-6]. To date around 90,000 distinct alternative splicing events, in human genes, have been predicted [7]. Even though a difference in prevalence of alternative splicing between vertebrates and invertebrate has been shown [8], several studies indicate that there is no difference in prevalence among different mammalian species [8,9]. However, results regarding the conservation of alternative splice events between mammalian species have been conflicting [9-11].

In addition to simply detecting the existence of alternative splice events from EST alignments, tissue annotation of ESTs can be used for *in silico *prediction of the expression pattern of alternative transcripts [12-14]. Alternative splicing of pre-mRNA, together with tissue-restricted gene transcription, allows cells in multicellular organisms to express the protein isoforms that are required for the specific function of each cell at different times and in different tissues. Tissue and temporal restriction of transcription and alternative splicing have similar requirements, as both use specialised mechanisms utilising nucleotide sequences and proteins [15]. Alternative splicing of pre-mRNA can contribute to the differences between tissues or cells either by regulating gene expression or creating proteins with various functions encoded by one gene.

Large scale EST detection of tissue-specific alternative splicing in the human transcriptome has shown that 10-30% of the alternatively spliced genes have evidence of tissue-specific splice isoforms [16]. However, interpretation of EST data should be made with caution, even when assuming that a wide variety of potential problems are carefully filtered out, as for example genomic contamination and incomplete mRNA processing [17]. Individual ESTs might represent rare splice forms or even errors made by the splicing machinery that do not constitute a significant fraction of transcript isoforms, or do not make a correctly functional protein. Both cDNA synthesis techniques and EST sequencing can give rise to incomplete mRNA coverage, which may bias our perception of splicing events. Furthermore, when alternative splice events are found, information about their tissue-specific regulation is often poor or unavailable [e.g. [12]].

Several databases of alternative splicing have been developed. In general, these databases can be divided into two categories, depending on the approach used. One category of database uses existing annotations of alternative splicing by data mining sequences and literature from databases such as EMBL, GenBank, Swiss-Prot and MedLine. This category includes 'Alternative Exon Database' [18], 'Alternative Splicing Database' [19] and 'EST Derived Alternative Splicing Database' [9]. Another category of database examines alignments between EST/cDNA sequences and genomic sequences, e.g. 'Putative Alternative Splicing Database' [20], 'The Alternative Splicing Annotation Project' [21] and 'Alternative Splicing and Transcript Diversity' [22]. All these databases differ in the size of the generated data sets, the methods employed and hence in their quality as a resource.

For more functionally predictive data, we suggest that conservative criteria should be applied when using EST data to predict tissue-specific alternative splice events. In this study, we have used sequence information from a set of cDNA libraries generated by the Sino-Danish Pig Genome Project [23]. This resource contains sequence data from 97 non-normalised libraries from 35 different tissues and three different developmental stages. This EST data set has been assembled together with publicly available porcine EST sequences in a database (PigEST), comprising more than one million EST sequences http://pigest.ku.dk/.

## Results

### In silico detection of alternative splicing in the pig

**Figure 1:**
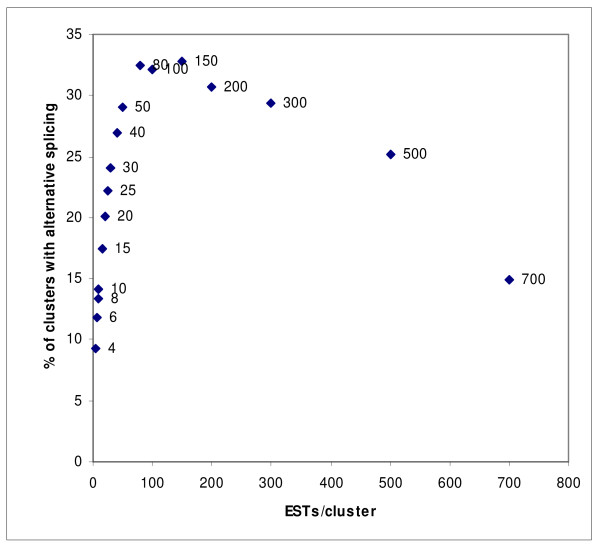
**Relationship between number of ESTs/cluster and percentage of clusters containing putative alternative splice events**. The number of ESTs in clusters containing alternative splice events (with four as the minimum number of EST in a cluster) is shown at the x axis. The numbers at the y axis represents the percentages of clusters containing alternative splice events compared to the total number of clusters. The numbers at the data points refer to the bin of cluster size.

The porcine database PigEST [23] contains 48,629 gene clusters, however only 27,654 of them contain four or more ESTs, which we require as a minimum for detecting alternative splicing according to the selection criteria set in the present study; as each splice variant has to be verified by at least two EST sequences. Putative alternatively spliced transcripts were detected in 2,515 clusters. Thus, disregarding cluster size we estimate that approximately 9% of the clusters in the PigEST database contain alternative splice variants. However, the relationship between number of ESTs in each cluster and the number of ESTs in clusters with alternative splicing indicate that approximately 30% of the porcine pre-mRNAs are subjected to alternative splicing (see Figure 1). Most clusters contain only two putative splice variants, but some clusters appear to have more than ten. The average number of putative splice variants in the porcine EST clusters containing alternative splicing is 2,9.

### Gene ontology

To address the issue of functional representations of the splice variants in each library, annotation using Gene Ontology (GO) was performed for each of the three main classes of GO; molecular function, cellular component and biological process [24]. The results of the GO analysis are shown in Additional File 1 and detailed information on the individual libraries is provided in Additional File 2. The GO analysis shows an increase of alternative splicing in the category "developmental and biological-process-unknown" in the adult muscle libraries. Also, the category "viral life cycle" is slightly overrepresented among the alternatively spliced clusters in the lung libraries. For cellular component there is an increase of alternative splicing in the category "extracellular matrix" in joint capsule, eye and skin related libraries. In libraries originating from embryonic tissues an increase of alternative splicing of "transcription-regulator-activity genes" is observed.

### In silico detection of tissue-specific alternative splicing in the pig

A total of 100 porcine genes with the most distinct tissue-specific alternative splicing were selected out of 2,515 EST clusters with highest confidence. The list (shown in the Additional File 3) was generated according to *in silico *analysis of the EST data using conservative criteria. Transcripts were only considered as an alternative splice variant if there were two or more ESTs that could confirm the transcript variant. Hence, only clusters with four or more transcripts were evaluated for alternative splicing. All clusters containing alternative splicing can be found in the PigEST Database at http://pigest.ku.dk/more/altsplice

### Validation of tissue-specific alternative splicing in pig

From the list of 100 putative tissue-specific alternatively spliced genes, 10 genes, selected randomly from the list, were validated experimentally with respect to the predicted alternative splice events (see Table 1). The transcript ratios for single alternative splice events were studied in seven of the genes; *ANAPC11, ATP5S, BSCL2, EIF4E2, IGF2, HNRPLL *and *PPP3CC *(for full gene names and cluster names see Additional File 2). In *CEP27 *both exon skipping of exon 5 and exon skipping of exons 4 and 5 together were examined. In the case of *RBM4*, two independent splice events were studied involving alternative 3' splice site of exon 3 and skipping of exon 5 as well as skipping of a part of exon 5. Finally, an exon skip of several exons as well as a 5' alternative splice event were studied in the *AUH *gene. Some of the genes are represented by additional putative alternative splice events, but since they were not predicted to be tissue-specific according to the *in silico *study they were not included in the experimental validation.

**Table 1:** Gene information on experimental verified splice variants.

	Gene			Tissue-specificity		
**No.**	**Symbol**	**Name**	**Function**	**EST**	**qPCR**	***P*-value**

4	IGF2	insulin-like growth factor 2 (somatomedin A)	Cell proliferation	Liver	Liver	5.5 × 10^-27^

7	EIF4E2	eukaryotic translation initiation factor 4E family member 2	Translation	Intestine	Intestine/lymph	2.6 × 10^-44^

14	RBM4	RNA binding motif protein 4	Transcription	Thymus Brain	Brain*	7.2 × 10^-36^ 1.9 × 10^-9^

15	ANAPC11	anaphase promoting complex subunit 11 homolog (yeast)	Cell cycle	Muscle, Lymph - Brain - Intestine	Liver	9.7 × 10^-5^

27	ATP5S	ATP synthase, H+ trans-porting, mitochondrial F0 complex, subunit s (factor B)	Ion transporter	Muscle	Muscle	5.7 × 10^-18^

28	HNRPLL	heterogeneous nuclear ribonucleoprotein L-like	RNA processing	Brain	Brain*	1.0 × 10^-21^

41	PPP3CC	protein phosphatase 3 (formerly 2B), catalytic subunit, gamma isoform	Phophatase	Brain	Muscle*	2.1 × 10^-42^

71	CEP27	centrosomal protein 27 kDa	Centrosome	Brain	Brain	7.8 × 10^-18^ 3.3 × 10^-15^

85	BSCL2	Bernardinelli-Seip congenital lipodystrophy 2 (seipin)	unknown	Ovary	Ovary*	1.7 × 10^-30^

97	AUH	AU RNA binding protein/enoyl-Coenzyme A hydratase	RNA catabolism	Heart	Heart* Ovary	2.0 × 10^-19^ 4.0 × 10^-16^

No. refers to the ranking on the predicted tissue-specific list (Additional File 2). Tissue specificity is listed both in regard to the *in silico *predictions (manual inspection of EST data) and in regard to the experimental verifications (qPCR). The P-value is referring to the ANOVA results. Tissues marked with * are not exclusive tissue-specific but tissue-specific ratios between the splice variants have been detected.

**Figure 2:**
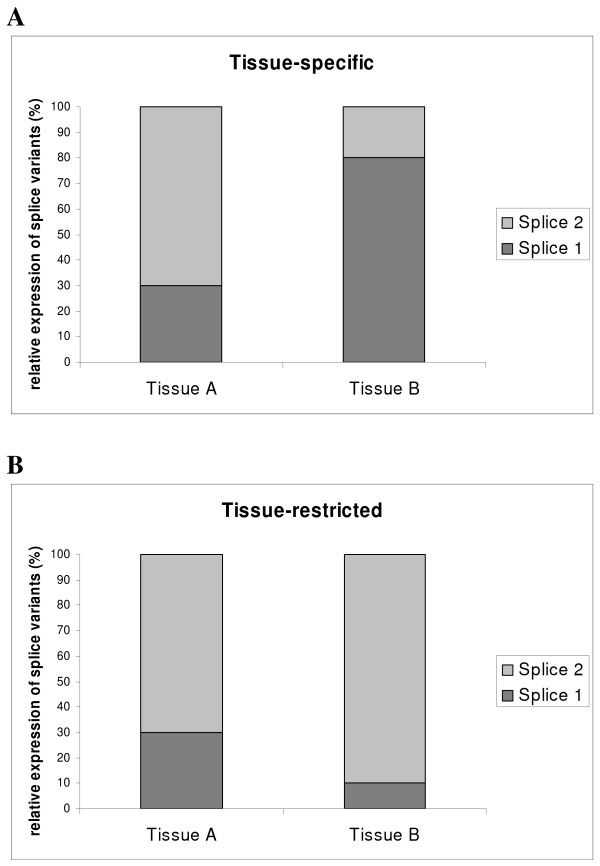
**This figure illustrates how tissue-specific and tissue-restricted expression pattern of two splice events have been defined in the study**. A) tissue-specific expression pattern is defined as one splice variant being dominant in some tissues whereas the other splice variant is dominant in other tissues. B) tissue-restricted expression is defined as expression pattern where the ratio between two splice variants is significantly different in different tissues, however always with the same splice variant as the dominant one.

In the present study, alternative splice-product ratios are used as a quantitative measure of gene expression in different tissues. Quantitative PCR (qPCR) amplification of the two alternative splice transcript forms of a gene can be used to calculate a ratio with good precision from steady-state RNA samples. Stringent tissue-specific alternative splice events were defined as events in which one splice variant was the major transcript of the gene in one or more tissues whereas the other splice variant was dominant in other tissues (illustrated in Figure 2A). In addition, the ratio between two splice variants was defined as tissue-restricted when one splice variant was significantly up- or down-regulated in relation to the other splice variant; however without one splice variant being directly tissue-specific (illustrated in Figure 2B).

**Figure 3:**
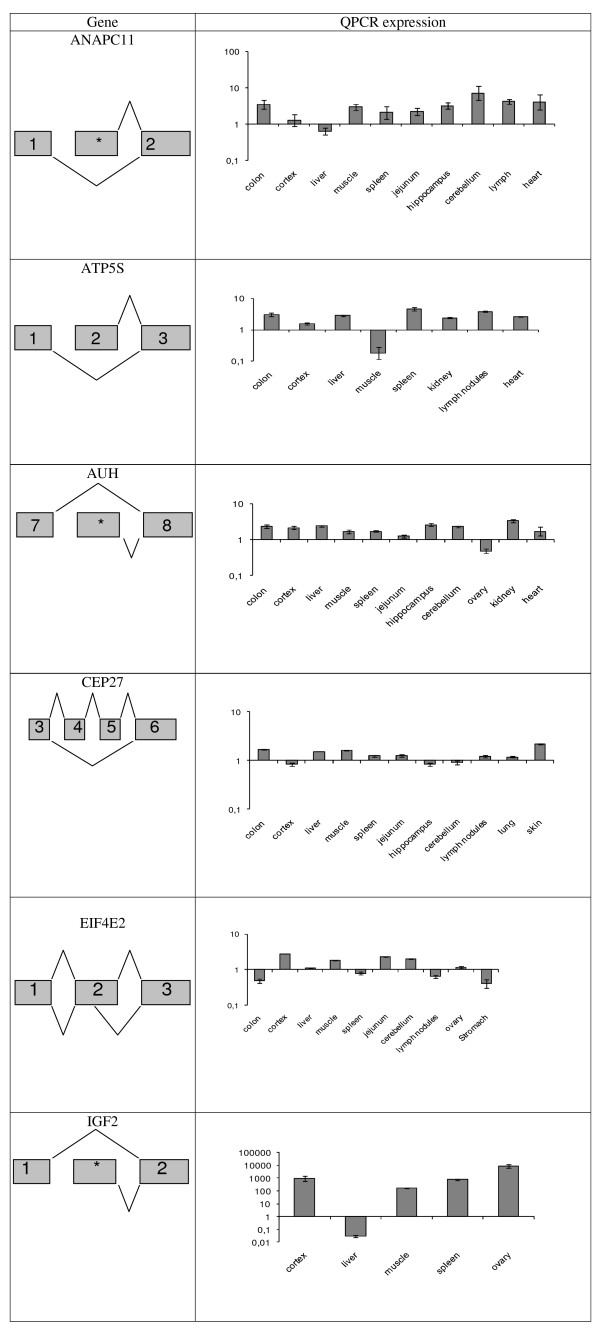
**Gene structure and QPCR data for genes with validated tissue-specific or tissue-restricted alternatively spliced variants**. QPCR data presented as changes in fold ratios between expressions of two splice variants in different adult pig tissues for the genes: *ANAPC11*, *ATP5S*, *AUH*, *CEP27*, *EIF4E2 *and *IGF2*. Data are normalised with a reference gene index. Values are geometric means ± Geometric S.E.M.; N = 4 animals. The highest expressed splice variant in the majority of the tissues is used as the numerator and the less expressed splice variant is used as the denominator. The highest expressed splice variant is shown in the upper part of the "Gene" column and the less expressed splice variant is showed in the lower part of the "Gene" column. *sequence that have not been defined as exon in any other species.

**Figure 4:**
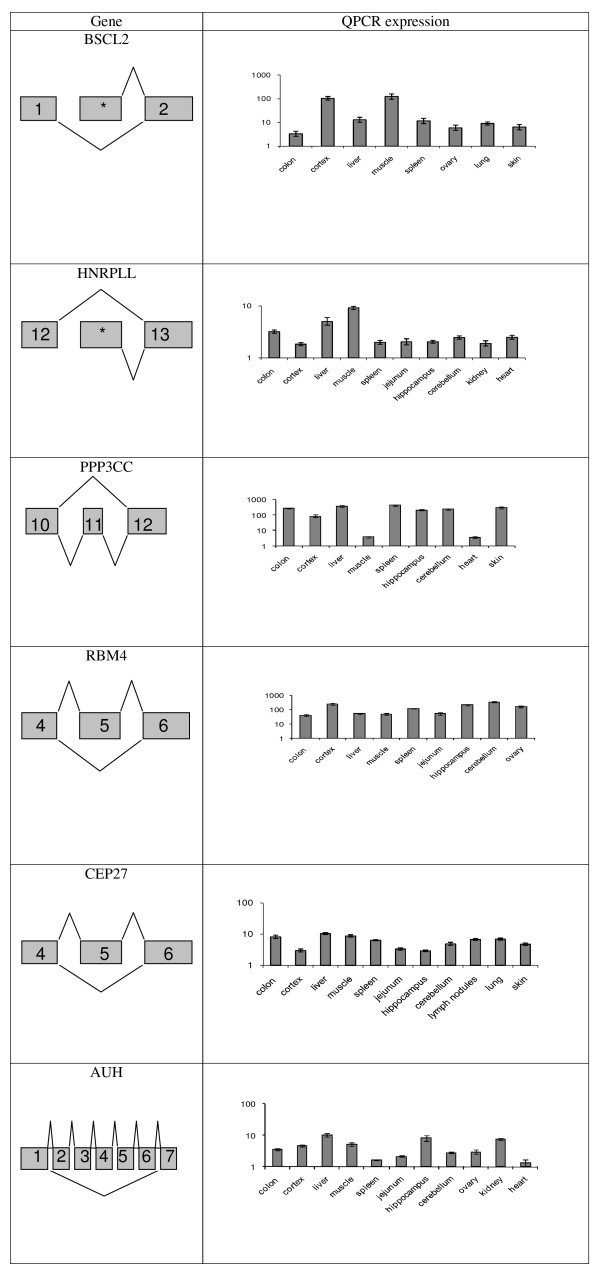
**Gene structure and QPCR data for genes that are neighed expressed in a tissue-specific manner nor in a tissue-restricted manner, however, with validated alternatively spliced variants**. QPCR data presented as changes in fold ratios between expressions of two splice variants in different adult pig tissues for the genes: *BSCL2*, *HNRPLL*, *PPP3CC *and *RBM4*. Data are normalised with a reference gene index. Values are geometric means ± Geometric S.E.M.; N = 4 animals. The highest expressed splice variant in the majority of the tissues is used as the numerator and the less expressed splice variant is used as the denominator. The highest expressed splice variant is shown in the upper part of the "Gene" column and the less expressed splice variant is showed in the lower part of the "Gene" column. *sequence that have not been defined as exon in any other species.

Comparisons of splice isoform ratios in different porcine tissues using qPCR showed that six genes (*i.e. ANAPC11*, *ATP5S*, *AUH*, *CEP27*, *EIF4E2*, *IGF2*) had stringent tissue-specific alternative splice events, whereas the remaining four genes (*i.e BSCL2*, *HNRPLL*, *PPP3CC*, *RBM4*) were not expressed in an exclusive tissue-specific manner, but statistically significant tissue-specific ratios between alternative splice variants were detected. Examples of qPCR results for splice events with tissue-specific and tissue-restricted alternative splice variants are shown in Figure 3 and Figure 4, respectively. The ratios between the alternative splice events in different porcine tissues were statistically tested using a two-way ANOVA with individual pigs as co-variable. The result of tissue-specificity is shown in Table 1. The majority of the variation between the splice variants was found to be a result of tissue-specific regulation. However, the two-way ANOVA showed that the individual pigs also contribute significantly to the variation with approximately one-fifth of the total variation (data not shown).

The tissues tested for all alternative splice events were colon, cortex, liver, muscle and spleen in addition to selected tissues relevant for the specific gene according to the *in silico *prediction of the tissue-specificity. Tissue-specificity or tissue-specific ratio of the splice variants were detected mostly in muscle/heart, intestine and brain, but two cases of liver-specific and one case of ovary-specific expression were also detected. The successful use of the conservative criteria for *in silico *prediction of splice events is reflected by the rate of experimental verification: all of the 16 candidate splice events studied experimentally were confirmed to be present at levels that indicate functional importance (see Table 1). Thus, in total five of the 16 alternative splice events studied (~31%) appear to be species specific. Cross-species studies using mouse or human ESTs have shown high sequence and gene structure similarity [4,25]. Alignment of the porcine splice variants with mouse and human genomic sequences showed several putative pig specific splice variants. The porcine *AUH *gene produces an alternative splice variant that is ovary specific. Moreover, no significant BLAST hit was found against expressed sequences in other species. *PPP3CC *has an exon skip with an exon not defined in human, but a highly conserved sequence of the exon was found in an intron of the orthologous human *PPP3CC *gene. Similarly, the porcine *BSCL2 *gene produces two different transcripts with distinct 5' initiation exon and one of the *BSCL2 *initiation exons seems to be putative pig specific, but with a highly orthologous sequence in the human *BSCL2 *gene assigned between exon 1 and 2. Two porcine genes, *ANAPC11 *and *IGF2*, also produce transcripts with different 5' initiation exon unique for pig and both of these transcript were found to be liver-specific. Thus, in total five of the 16 alternative splice events studied (~31%) appear to be species specific.

## Discussion

The number of clusters with alternative splicing increases with the number of ESTs per cluster as shown in Figure 1, but attains a maximum between 50-300 ESTs per cluster. Approximately one-third (30%) of the clusters with 50-300 ESTs contain alternative splice variants. This estimate is based on the assumption that the number of ESTs and hence the coverage of the individual gene is more important than expression level when the aim is to identify alternative splicing. A decrease is observed in clusters with more than 300 ESTs, most likely explained by the low number of clusters in this category. The results indicate that a cluster size of at least 50 ESTs is optimal for detection of alternative splicing. The estimated 30% porcine genes with alternative splicing are in agreement with large-scale estimations of alternative splicing data based on EST predictions in human and mouse. Human EST comparisons showed that 35% [5] and 38% [1] of the human genes had alternative splicing and in mouse the frequency of alternative splicing was estimated to 33% of the genes [26]. It is, however, important to note that direct comparison between studies is difficult since stringency of prediction may differ, e.g. using two versus one EST to predict an alternative splice event and/or use of different gap size. More recent studies of alternative splicing estimate a larger proportion of human genes to have alternative splice variant [3,4,27]. These studies are either based on a combination of genomics sequence information and EST data, or contain a greater amount of ESTs. In addition, supplementary cDNA libraries from more developmental and pathological stages will probably increase the number of observed alternative splicing events in porcine genes. We note that in human and mouse, where alternative splicing is estimated to be most prevalent, many cDNA libraries are cancer related and hence many of the alternative splice variants that were detected may have been pathological [28,29]. In contrast, most porcine cDNA libraries are constructed from healthy tissue [24,25,30-33].

Since our study is based on the use of EST consensus sequences as well as sub-cluster construction with at least two EST sequences, the reliability of the prediction of alternative splicing is high. The consensus sequences reconstruct parts of transcripts from multiple EST sequences and so contain a lower number of sequencing errors than the original EST data. Since consensus sequences have been aligned to reveal putative splice sites fewer splice site artefacts have been identified compared to studies based only on EST alignments. The successful use of the conservative criteria for *in silico *prediction of splice events is reflected by the rate of experimental verification: all of the 16 candidate splice events studied experimentally were confirmed to be present at levels that indicate functional importance.

In total five of the 16 alternative splice events studied (~31%) appear to be species specific. These findings are comparable to human and rodent studies where it has been estimated that 25% of human alternatively spliced exons are not present in the orthologous murine genes [9,34]. These studies also show that approximately half of the alternatively spliced genes produce species-specific isoforms. In addition, even more alternative splice events that cannot be defined as species-specific have been shown to be regulated in a species-specific manner [35]. It has been hypothesised that alternative splicing can be used for creation of new exons and hence novel proteins, since the majority of new exons have unique sequences in the genome, suggesting that most new exons might originate from intronic sequences [36]. This is supported by a comparison between human-mouse-rat alternative splicing patterns suggesting that alternative splicing is associated with a large increase in the frequency of recent gain or loss of exons [35]. Thus, it seems that species-specific alternative splicing and species specific regulation of expression provide important contributions to speciation.

## Conclusion

In accordance with human and rodent studies we estimate that approximately 30% of the porcine genes undergo alternative splicing. We found a good correlation between EST predicted tissue-specificity and experimentally validated splice events in different porcine tissue. This study indicates that a cluster size of around 50 ESTs is optimal for *in silico *detection of alternative splicing. Although based on a limited number of splice events, the study supports the notion that alternative splicing provides an important impact on species differentiation since 31% of the splice events studied appears to be species specific.

## Methods

### In silico data preparation

In total 2515 porcine genes (clusters) with putative alternative splice events were predicted using porcine EST sequences available in the PigEST database and analysed with the Distiller software as described by Gorodkin *et al*. [23]. The ESTs assembled in a cluster were divided into consecutive 12-mers by the consensus builder of Distiller. The two consecutive 12-mers, taking SNPs into account, were compared to detect variants [37]. A transcript is considered as an alternative splice variant if there are two or more ESTs that can confirm the transcript variant. Hence, only clusters with four or more transcripts can be evaluated for alternative splicing using this criterion. The Distiller software will detect putative exon skips, 3' and 5' alternative splicing as well as other rare splice categories, but cannot give information on the actual splice type, although gaps less than 40 bp are not detected as alternative splicing. Each of the putative candidate genes can contain two or more different splice variants.

To obtain information of Gene Ontology (GO) on clusters containing alternative splicing, GO analysis (UniProt 36.0 release) were performed for each of the three main classes; molecular function, cellular component and biological process [24]. We are aware that GO categories have been updated since the study by Gorodkin *et al*. [23] was performed, however, for comparison the same categories were applied in this work. GO expression profiles were constructed as followed; the expressions of alternative splicing of each of the categories were accumulated for clusters containing alternative splicing and normalised into fractions. For each library a log odds ratios of the GO categories between the actual fractions and the average fractions of clusters labelled with alternative splicing were computed. A heat map of the log odds values (in bits) for each of the GO categories was constructed. This was done by comparing the actual fraction of the GO categories with the respective average fractions of ESTs from clusters labelled as alternative splicing.

EST sequences are assembled into clusters and divided into sub-cluster representing different putative splice variants. One hundred clusters with evidence of tissue, in form of library, specific alternative splice event were generated based on the 2515 genes with presumed alternative splicing. The selected clusters contain at least one pair of transcripts that satisfy the following criteria; 1) pairs of transcripts from the same cluster (parent cluster) where no more than 10% of the clones in either sub-cluster come from the same libraries as the other sub-cluster 2) the best sub-cluster pairs were determined to be for pairs where the smaller of the two sub-clusters contain the highest fraction of the total ESTs. The consensus sequences for both splice variants were used for comparison with genomic porcine sequences, if available, to reveal the exon-intron structure. When no genomic porcine sequence was available, comparison of the pig sequence with human and mouse genomic sequences was used to obtain the gene structure, under the assumption that the exon-exon boundaries are conserved between human, mouse and pig.

### Primer design

All the primers were designed using the Primer 3 software [38]. The primers were designed to span one splice site also candidate for an alternative splice site, an approach which minimise inaccuracies in quantification due to genomic DNA contamination. The secondary structure of the amplicons were analyzed by Mfold using the criteria -3<dG<0 [39] to optimize the PCR efficiency. Primers and amplicons were *in silico *verified with BLASTN for specificity and the size of the PCR products were confirmed by gel electrophoresis and melting point analysis during qPCR. Information of primer sequences and sub-cluster names is found in Table 2.

**Table 2:** Sub-cluster names (from the PigEST database) and primer sequences of experimentally verified splice variants.

Gene symbol	Sub-cluster name	Forward primer (5'→3')	Reverse primer (5'→3')	Amplicon length (bp)
IGF2	Ss1.1-Utr1-UTR01C050081.5SS	AGCCCACAGCGATTCCAA	AGTCTCACTGGGGCGGTAAG	110

	Ss1.1-Liv1-LVR010049B06.5SS	CCGGCTTCCAGATTCCAA	AGTCTCACTGGGGCGGTAAG	143

EIF4E2	Ss1.1-Pig3-SRG8020J01.3SS	CCGGTTTCAGGAGGACATTA	GAAAGCTTGAATTGTCCTTGATG	164

	Ss1.1-Tes1-TES01A030013.5S	CCGGTTTCAGGAGGACATTA	ACTCCTCCCAGGCCTTGAT	161

RBM4	Ss1.1-Lng1-LNG010007H10.5SS	TCGGCATTGCGCGGCTGT	CTCGAACAGTGAGCGGATCT	94

	Ss1.1-Utr1-UTR010079B01.5S	GATTTTCGTGCGGGCTGT	CTCGAACAGTGAGCGGATCT	94

	Ss1.1-Lng1-LNG010007H10.5SS	CATCAGAGGCCTTGACAACA	CGGCAACAGGTGTCGATAG	101

	.1-Thy1-THY010092E11.5SS	TCGTGAAAGATTATGCCTTCG	CACACACATCCCACCTTGAA	101

	Ss1.1-Utr1-UTR010079B01.5SS	TCGTGAAAGATTATGCCTTCG	CTCTTTCCCGCACCGATAGC	124

ANAPC11	Ss1.1-Pig4-TMW8023A04.5SS	GTGGGCCTTGCAGGAGTT	GAGCCACTTGAGGATGCAGT	246

	Ss1.1-rnca27b_a11.5S	CCGCGGTCGTTTATATACCT	GAGCCACTTGAGGATGCAGT	276

	Ss1.1-Pig3-SRG8012J07.3S	TGGCTCCCAAGTGCTGTAG	GAGCCACTTGAGGATGCAGT	267

ATP5S	Ss1.1-ruio31_j9.5SS	TCCCTTCGACGCTATCAAAT	GATGCGTTCATGATCCACTTT	144

	Ss1.1-Mixn-0026b16.5SS	ACCAAGTGGCAAATCAAATG	GATGCGTTCATGATCCACTTT	143

HNRPLL	Ss1.1-rret19_c4.5S	GATAAGAGTTCCCAATGGTTCC	GGTAAACAAATTCTAACATGCTCTTC	101

	Ss1.1-rpbfe0126_n23.5S	TGAAAGTTTGATGAAGGGAACT	GGTAAACAAATTCTAACATGCTCTTC	136

PPP3CC	Ss1.1-LyN1-MLN01A040100.5	GATGAAATTGAAGGAGGCACT	TCCCGAAGAATTGAAAAGACC	98

	Ss1.1-Mixn-0001e03.5S	AAGCTATCAGAAAGGAGGCACT	TCCCGAAGAATTGAAAAGACC	99

CEP27	1Ss1.1-Liv1-LVRM10031B03.5SS	CTAAAGAAAATGAGCACAGC	TCTGTTGTTCTCGCCACTTG	97

	2Ss1.1-rsug28_c19.5S	GCTGTTTATCACAGAGCACAGC	TCTGTTGTTCTCGCCACTTG	99

	3Ss1.1-Pig4-TMW8033B03.3S	CCTTCATTTTTGGAGCACAG	TCTGTTGTTCTCGCCACTTG	98

BSCL2	Ss1.1-Thy1-THY010012C06.5	GGTGTGCAGAGACCAGATCA	GGTTCAGGCCTTGAGTTCCT	117

	Ss1.1-Spl1-SPL010003H07.5S	GCTGCTCGCTCTGAGTCC	GGTTCAGGCCTTGAGTTCCT	111

AUH	Ss1.1-Ova1-OVR010018B04.5SS	CTACAGCTCTGAGGCGAAGA	CACCACAATTCCTCGGTTCT	76

	Ss1.1-Liv1-LVR010068B01.5S	GGCAATTATTCCTGGTGGAG	TCAAATGTTGCCTTTTCAGC	107

	Ss1.1-Pig3-SRG8009K09.3S	TGTCTCCTGTGCTGCAGTATG	TGTGTGAGGGTACATGTGTGAA	108

### Tissue collection and RNA extraction

Nineteen different porcine tissues were collected from four (2 females and 2 males) 3 months old siblings (Danish crossbreds). Ovaries were collected from four 6-months old female crossbreds. Total RNA was extracted in duplicate for each tissue and animal using different protocols depending on the tissue according to each manufacturer protocol: TRIreagentR (Molecular Research Centre, inc.) for stomach, lung, heart, kidney, lymph node, colon, jejunum, illeum, duodenum, cartillage, spleen and skin. RNeasy Lipid Tissue Kit (Qiagen) for adipose (subcutaneous), cortex cerebri, cerebellum, hippocampus and ovary. RNeasy Fibrous Tissue Kit (Qiagen) for muscle (longissimus dorsi). RNeasy maxi Kit (Qiagen) for pancreas. RNeasy mini Kit (Qiagen) for liver. Contaminating genomic DNA was degraded by treatment with RQ1 RNase-free DNase (Promega) according to the instructions manual, followed by spin-column purification (Qiagen RNeasy mini or micro cleanup kit). The total RNA was quantified by optical density and the quality was evaluated by rRNA 28S/18S bands inspection by gel electrophoresis and by RIN number (2100 Bioanalyzer, Agilent technologies). RIN of 5 was considered minimum acceptable.

### cDNA synthesis

Four μg of DNase-treated total RNA were reverse transcribed at 42°C using Improm-II™ reverse trancriptase (Promega) and a 3:1 mixture of Random hexamers and Oligo-dT, according to the manufacturers recommendations. Prior to use in qPCR, cDNA was diluted 1:16 with H_2_O. The panel consists of four biological replicates and four experimental replicates for each tissue, in total 8 samples/tissue.

### Quantitative PCR

Real-time RT-PCR was performed on a Mx3000 detection system (Stratagene). For each transcript as well as for the reference genes, a serial of dilutions of the purified PCR product generated for each specific primer pair was done in order to build a standard curve. Single reactions were prepared for each cDNA and for each dilution. Each PCR also included a reverse transcription negative control to confirm the absence of genomic DNA, a non template negative control to check for primer dimers and a porcine genomic DNA control to verify no amplification of genomic DNA with the primers. Briefly, 5 μL QuantiFast SYBR Green PCR Master Mix (Qiagen, Hilden, Germany) was mixed with 5 pmol of each primer and 2 μL of diluted cDNA or water or dilution in PCR plates (AB Gene, ThermoScientific, Waltham, MA) and underwent PCR amplification (model Mx3000P, Stratagene, La Jolla, CA). The cycling conditions were one cycle of denaturation at 95°C/5 min, followed by 40 two-segment cycles of amplification (95°C/10 sec, 60°C/30 sec). Fluorescence was measured automatically during amplification and one three-segment cycle (95°C/1 min, 60°C/30 sec, 95°C/30 sec) was performed to obtain the melting curve for the product. The baseline adjustment method of the thermocycler (Mx3000, Stratagene) software was used to determine the quantities in each reaction. All samples were amplified and the mean was used for further analysis. Quantity values were normalised by a reference index constructed from four reference genes; using *YWHAZ*, *TBP*, *RPL4 *and *BACT*. The program geNorm [40] was used for calculation of the reference index as well as for selection of the four most suitable reference genes from a panel of nine potential reference genes [41]. The specificity of amplification was confirmed by DNA sequencing of the PCR products. The amplified PCR products were purified using QIAquick PCR Purification Kit (Qiagen) and sequenced using BigDye^® ^Terminator v3.1 Cycle Sequencing Kit (Applied Biosystems) on an ABI PRISM^® ^3130 Genetic Analyzer.

### Data analysis/statistical analysis

Changes in fold ratio of mRNA expression between the two splice variants was calculated as relative quantity values using the formula;  where E_splice1 _is the efficiency of qPCR for splice variant 1 and E_splice2 _for splice variant 2. Ct_splice1 _and Ct_splice2 _are the Ct values for each sample. The efficiencies were calculated based on the standard curve. The fold ratio change was LOG transformed according to Gallup and Ackermann [42] to generate normal distribution and the data was tested for statistical significance of differential mRNA expression between the splice variants using two-way ANOVA with tissue as well as pig individual and sex as co variables. T-test was performed if the main effect was significant.

N = 4 was calculated as a mean of the two biological replicates from each animal. A *P *value of < 0.05 was considered statistically significant. The geometric means and the geometric standard error of the mean (S.E.M) were calculated.

## Competing interests

The authors declare that they have no competing interests.

## Authors' contributions

ABN has been responsible for the experimental work and made substantial contributions to conception and design of the study. Furthermore, she has been involved in drafting the manuscript. SC has been responsible for RNA work and involved in data analysis, drafting the manuscript and revising it critically for important intellectual content. MJG has made substantial contributions to conception and design of the study. CBJ has made substantial contributions to conception and design of the study and been involved in drafting the manuscript and revising it critically for important intellectual content. JG has made GO analysis and performed bioinformatics support. MF has made substantial contributions to conception and design of the study. She has been involved in drafting the manuscript and revising it critically for important intellectual content, and given final approval for the version to be published.

## Supplementary Material

Additional file 1 **Gene Ontology of clusters with predicted alternative splicing**. Figure 1A-C: Gene Ontology of clusters with predicted alternative splicing. For information on the specific tissues used for library construction see Additional file 2 (Table 1). Click here for file

Additional file 2 Table 1: Description of the libraries that the Gene Ontology clusters have been constructed from. Click here for file

Additional file 3 Table 2: List of 100 alternative spliced porcine genes predicted tissue-specificity. Click here for file
