# Supplementary material for: A study of alternative splicing in the pig
Source: BMC Res Notes. 2010 May 5;3:123. doi: 10.1186/1756-0500-3-123 (PMC2882375; doi:10.1186/1756-0500-3-123)
Supplement: Additional file 1 — Gene Ontology of clusters with predicted alternative splicing. Figure 1A-C: Gene Ontology of clusters with predicted alternative splicing. For information on the specific tissues used for library construction see Additional file 2 (Table 1). [file 1756-0500-3-123-S1.PPT]

## Slide 1
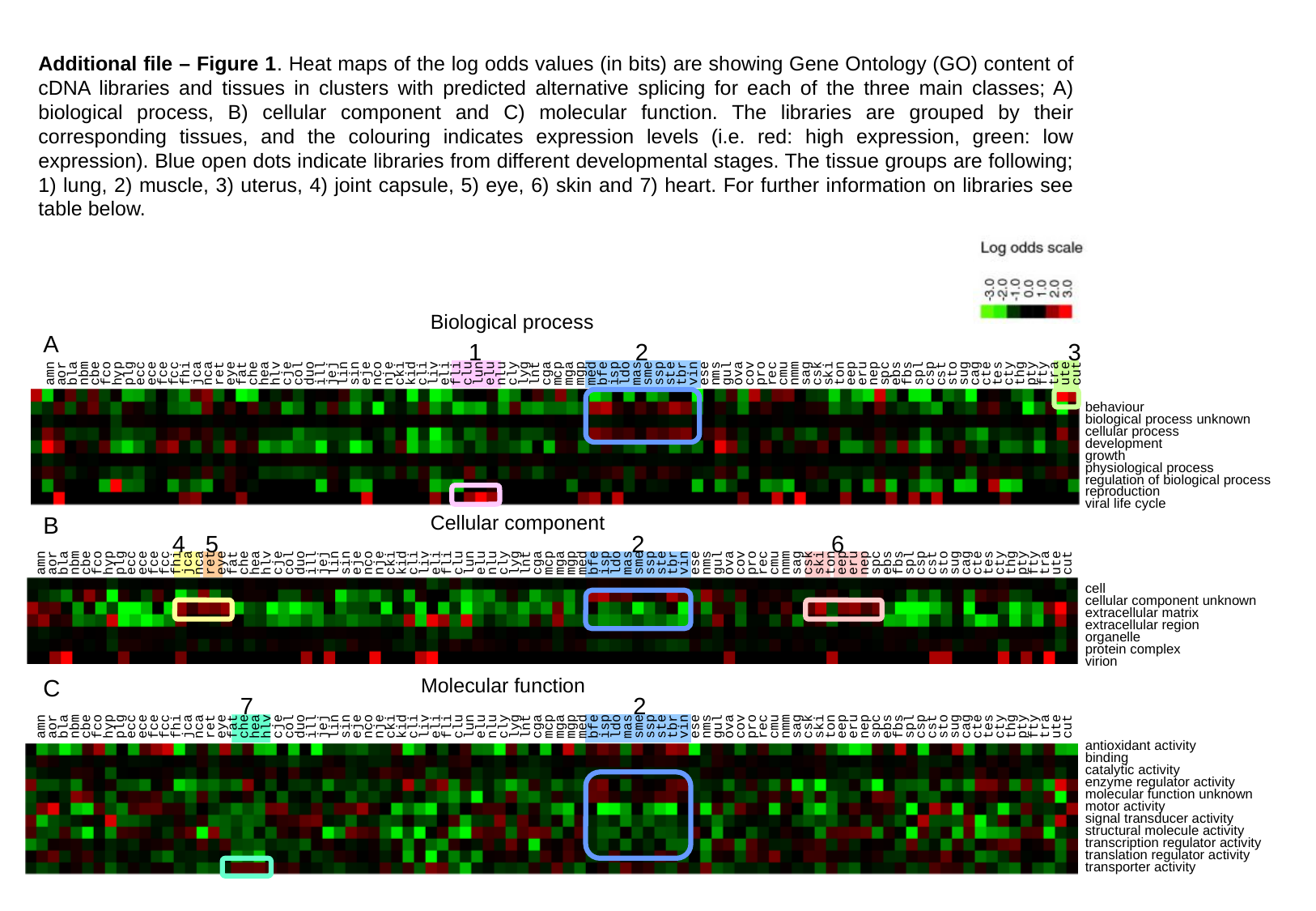

Additional file – Figure 1. Heat maps of the log odds values (in bits) are showing Gene Ontology (GO) content of cDNA libraries and tissues in clusters with predicted alternative splicing for each of the three main classes; A) biological process, B) cellular component and C) molecular function. The libraries are grouped by their corresponding tissues, and the colouring indicates expression levels (i.e. red: high expression, green: low expression). Blue open dots indicate libraries from different developmental stages. The tissue groups are following; 1) lung, 2) muscle, 3) uterus, 4) joint capsule, 5) eye, 6) skin and 7) heart. For further information on libraries see table below.
amn
aor
bla
nbm
cbe
fco
hyp
plg
ecc
ece
fce
fcc
fhi
jca
nca
ret
eye
fat
che
hea
hlv
cje
col
duo
ill
jej
lin
sin
eje
nco
nje
cki
kid
cli
liv
eli
fli
clu
lun
elu
nlu
cly
lyg
lnt
cga
mcp
mga
mgp
med
bfe
isp
ldo
mas
sme
ssp
ste
tbr
vin
ese
nms
gul
ova
cov
pro
rec
cmu
nmm
sag
csk
ski
ton
eep
eru
nep
spc
ebs
fbs
spl
csp
cst
sto
sug
cag
cte
tes
cty
thg
pty
fty
tra
ute
cut
Biological process
A
1 2 3
behaviour
biological process unknown
cellular process
development
growth
physiological process
regulation of biological process
reproduction
viral life cycle
cell
cellular component unknown
extracellular matrix
extracellular region
organelle
protein complex
virion
antioxidant activity
binding
catalytic activity
enzyme regulator activity
molecular function unknown
motor activity
signal transducer activity
structural molecule activity
transcription regulator activity
translation regulator activity
transporter activity
Cellular component
B
4 5 2 6
amn
aor
bla
nbm
cbe
fco
hyp
plg
ecc
ece
fce
fcc
fhi
jca
nca
ret
eye
fat
che
hea
hlv
cje
col
duo
ill
jej
lin
sin
eje
nco
nje
cki
kid
cli
liv
eli
fli
clu
lun
elu
nlu
cly
lyg
lnt
cga
mcp
mga
mgp
med
bfe
isp
ldo
mas
sme
ssp
ste
tbr
vin
ese
nms
gul
ova
cov
pro
rec
cmu
nmm
sag
csk
ski
ton
eep
eru
nep
spc
ebs
fbs
spl
csp
cst
sto
sug
cag
cte
tes
cty
thg
pty
fty
tra
ute
cut
Molecular function
C
 7 2
amn
aor
bla
nbm
cbe
fco
hyp
plg
ecc
ece
fce
fcc
fhi
jca
nca
ret
eye
fat
che
hea
hlv
cje
col
duo
ill
jej
lin
sin
eje
nco
nje
cki
kid
cli
liv
eli
fli
clu
lun
elu
nlu
cly
lyg
lnt
cga
mcp
mga
mgp
med
bfe
isp
ldo
mas
sme
ssp
ste
tbr
vin
ese
nms
gul
ova
cov
pro
rec
cmu
nmm
sag
csk
ski
ton
eep
eru
nep
spc
ebs
fbs
spl
csp
cst
sto
sug
cag
cte
tes
cty
thg
pty
fty
tra
ute
cut
