# Supplementary material for: A study of alternative splicing in the pig
Source: BMC Res Notes. 2010 May 5;3:123. doi: 10.1186/1756-0500-3-123 (PMC2882375; doi:10.1186/1756-0500-3-123)
Supplement: Additional file 2 — Table 1: Description of the libraries that the Gene Ontology clusters have been constructed from. [file 1756-0500-3-123-S2.DOC]

**Additional file Table 1.** From Gorodkin *et al.* 2007. The column 'Lib name' gives three letter code for the library. 'Tissue' indicates the overall tissue the library was generated from, where '(Animals)' indicates whether the library was generated from a single (S or B) or multiple (M) animals. Libraries listed with (M) and (S) represent the pig breeds (mostly cross-breeds) used in Danish breeding (Landrace, Yorkshire, Duroc, and Hampshire), whereas the libraries listed with (B) present Chinese pig breeds. 'Description' provides a short description. Library names beginning with 'C' originates from Chinese pig breeds (except for 'Col' and 'Cbe'), whereas the remaining libraries originated from Danish pig breeds.

| **Lib name** | **Tissue (Animals)** | **Description** |
| --- | --- | --- |
| Amn | Amnion (S) | - |
| Aor | Aorta (M) | - |
| Bla | Bladder (M) | - |
| Nbm | Bone marrow (S) | 115 days, bone marrow |
| Cbe | Brain (M) | Cerebellum |
| Cbr | Brain (B) | Brain (cortex) |
| Fco | Brain (M) | Frontal cortex |
| Hyp | Brain (S) | Hypothalamus |
| Pgl | Brain (M) | Pituitary gland |
| Ecc | Brain (S) | F 50 days, cortex |
| Ece | Brain (S) | F 50 days, cerebellum |
| Fce | Brain (S) | F 100, cerebellum |
| Fcc | Brain (S) | F 107, cortex cerebri |
| Fhi | Brain (S) | F 107 Hippocampus |
| Cbl | Haemopoetic (B) | Blood |
| Jca | Cartilage (S) | Joint capsule |
| Nca | Cartilage (S) | 115 days, cartilage |
| Pan | Endocrine glands (M) | Pancreas |
| Ret | Eye (M) | Retina |
| Eye | Eye (S) | F 50, eye |
| Fat | Fat (M) | Fat |
| Che | Heart (B) | - |
| Hea | Heart (M) | - |
| Hlv | Heart (S) | Left ventricle |
| Cje | Intestine (B) | Jejunum |
| Col | Intestine (S) | Large intest, colon asc. |
| Duo | Intestine (S) | Small intest, duodenum |
| Ill | Intestine (S) | Small intest, illeum |
| Jej | Intestine (S) | Small intest, jejunum |
| Lin | Intestine (M) | Large intestine |
| Sin | Intestine (M) | Small intestine |
| Eje | Intestine (S) | F 50, Jejunum |
| Nco | Intestine (S) | 115 days, colon |
| Nje | Intestine (S) | 115 days, jejunum |
| Cki | Kidney (B) | - |
| Kid | Kidney (M) | - |
| Cli | Liver (B) | - |
| Liv | Liver (M) | - |
| Eli | Liver (S) | F 50, liver |
| Fli | Liver (S) | F 100, liver |
| Clu | Lung (B) | - |
| Lun | Lung (M) | - |
| Elu | Lung (S) | F 50 days, lung |
| Nlu | Lung (S) | 115 days, lung |
| Cly | Lymphatic gland (B) | - |
| Lyg | Lymphatic gland (M) | - |
| Lnt | Lymphatic gland (S) | - |
| Cga | Mammary gland (B) | - |
| Mcp | Mammary gland (S) | Mammae, collostrum prod |
| Mga | Mammary gland (M) | 7 days after weaning |
| Mgm | Mammary gland (M) | 14 days after birth |
| Mgp | Mammary gland (M) | 7 days pre-birth |
| Med | Mediastinum (S) | - |
| Bfe | Muscles (M) | M. biceps femoris |
| Ctl | Muscles (B) | Tenderloin |
| Isp | Muscles (M) | M. infraspinatus |
| Ldo | Muscles (M) | M. longissimus dorsi |
| Mas | Muscles (S) | M. masseter |
| Sme | Muscles (M) | M. semimembranosus |
| Ssp | Muscles (M) | M. supraspinatus |
| Ste | Muscles (M) | M. semitendinosus |
| Tbr | Muscles (M) | M. triceps brachii |
| Vin | Muscles (M) | M. vastus intermedius |
| Ese | Muscles (S) | F 50, M. semitendinosus |
| Nms | Muscles (S) | 115 days, M. semitendinosus |
| Gul | Oesophagus (M) | - |
| Ova | Ovary (M) | - |
| Cov | Ovary (S) | - |
| Pla | Placenta (M) | - |
| Pro | Prostata (M) | - |
| Rec | Rectum (M) | - |
| Cmu | Rhinal mucosal membrane (B) | - |
| Nmm | Rhinal mucosal membrane (S) | 115 days, mucosal memb. |
| Sag | Salivary gland (M) | - |
| Csk | Skin (B) | - |
| Ski | Skin (M) | - |
| Ton | Skin (S) | Tip of tongue, mucosa |
| Eep | Skin (S) | F 50, epidermis |
| Eru | Skin (S) | F 50, regium bilicalis |
| Nep | Skin (S) | 115 days, epidermis |
| Spc | Spinal cord (M) | Spinal cord |
| Ebs | Spinal cord (S) | F 50 days, brainstem |
| Fbs | Spinal cord (S) | F 107 brainstem |
| Spl | Spleen (M) | - |
| Csp | Spleen (B) | - |
| Cst | Stomach (B) | - |
| Sto | Stomach (M) | - |
| Sug | Suprarenal glands (M) | - |
| Cag | Suprarenal glands (B) | Adrenal gland |
| Cte | Testicle (B) | - |
| Tes | Testicle (M) | - |
| Cty | Thyroid glands (B) | - |
| Thg | Thyroid glands (M) | - |
| Pty | Thyroid glands (S) | Piglet 2 days, thymus |
| Fty | Thyroid glands (S) | F 100, thymus |
| Tra | Trachea (M) | - |
| Ute | Uterus (S) | - |
| Cut | Uterus (B) | - |
